# Recurrent Acute Disseminated Encephalomyelitis (ADEM) after COVID-19-vaccination and after subsequent COVID-19-infection: A case report (part II)

**DOI:** 10.3389/fneur.2023.1149612

**Published:** 2023-03-10

**Authors:** Khouloud Poli, Markus Kowarik, Klaus Hamprecht, Thomas Iftner, Ulrike Ernemann, Ulf Ziemann, Sven Poli

**Affiliations:** ^1^Department of Neurology and Stroke, Eberhard-Karls University, Tübingen, Germany; ^2^Hertie Institute for Clinical Brain Research, Eberhard-Karls University, Tübingen, Germany; ^3^Institute of Medical Virology and Epidemiology of Viral Diseases, Eberhard-Karls University, Tübingen, Germany; ^4^Department of Diagnostic and Interventional Neuroradiology, Eberhard-Karls University, Tübingen, Germany

**Keywords:** recurrent, ADEM, COVID-19, SARS-CoV-2 antigen-specific IgG, CSF, biomarkers

## Abstract

Acute disseminated encephalomyelitis (ADEM) is an autoimmune disorder of the central nervous system (CNS), which is commonly associated to previous viral infection or immunization. Cases of ADEM with a potential relationship to both severe acute respiratory syndrome coronavirus 2 (SARS-CoV-2) infection and vaccination have been reported. We recently published a rare case of a 65-year-old patient who suffered from a corticosteroid- and immunoglobulin-refractory multiple autoimmune syndrome including ADEM following Pfizer-BioNTech coronavirus disease (COVID)-19 vaccination, and whose symptoms largely resolved after repeated plasma exchange (PE). Four months later, the patient was diagnosed with SARS-CoV-2 omicron variant infection after experiencing mild upper respiratory tract symptoms. Few days later, the patient developed severe tetraparesis with magnetic resonance imaging (MRI) showing multiple new inflammatory contrast-enhancing lesions in the left middle cerebellar peduncle, cervical spinal cord, and ventral conus medullaris. Repeated cerebrospinal fluid (CSF) analyses indicated blood-brain barrier damage (increased albumin ratio) without signs of SARS-CoV-2 invasion (mild pleocytosis, no intrathecal antibody production). SARS-CoV-2 specific immunoglobulin G (IgG) were detected in serum and to a much lower degree in CSF with close correlation between both concentrations over time, reflecting antibody dynamics of vaccine- and infection-induced immune response, and blood-brain barrier patency. Daily PE therapy was initiated. Given the patient's lack of improvement after seven PE, treatment with rituximab was considered. After a first dose, however, the patient suffered epididymo-orchitis leading to sepsis, and declined rituximab continuation. At 3-months follow-up, clinical symptoms had dramatically improved. The patient regained walking ability without assistance. This case of recurrent ADEM after COVID-19-vaccination and after subsequent COVID-19-infection strongly supports the hypotheses of neuroimmunological complications in these conditions being promoted by a systemic immune response and mediated by molecular mimicry of, both, viral and vaccine SARS-CoV-2 antigens and CNS self-antigens.

## Introduction

Acute disseminated encephalomyelitis (ADEM) is a rare immune-mediated inflammatory demyelinating disease of the central nervous system (CNS), which mainly affects children and occurs after viral infections or less often after vaccinations (1). Cases providing clinical evidence for ADEM or ADEM-like severe inflammatory disorder of the CNS in adults after severe acute respiratory syndrome coronavirus (SARS-CoV)-2-infection (2, 3) as well as SARS-CoV-2-vaccination (4, 5) have been described. Nevertheless, pathogenic mechanisms of CNS involvement remain unclear in both cases due to the diversity of neurological symptoms, laboratory and imaging findings, the variability of the clinical course, and the severity of infection, as well as the lack of comprehensive immunological, virologic, and epidemiological screening of neurosymptomatic patients with active coronavirus disease (COVID)-19 or after COVID-19 vaccination. Several mechanisms have been hypothesized to contribute to the neurovirulence of SARS-CoV-2 including virus invasion into the CNS, dysregulated systemic inflammatory response, hypoxia, and autoimmune reactions (6). Autoimmune reactions due to molecular mimicry and potentially enhanced by vaccine adjuvants are the presumed cause of vaccine-induced CNS disorders (7). We recently reported a case with messenger ribonucleic acid (mRNA) COVID-19-vaccine-induced multiple autoimmune syndrome including ADEM, myasthenia gravis, and thyroiditis, whose symptoms largely resolved after repeated plasma exchange (PE) (8). The same patient developed recurrent ADEM after SARS-CoV-2 omicron variant infection 4 months later.

## Case description

Two weeks after polymerase chain reaction (PCR)-confirmed diagnosis of SARS-CoV-2 infection with mild upper respiratory tract symptoms, a 65-year-old male patient developed muscle weakness and numbness in all extremities. The patient was admitted at a peripheral hospital and was referred to our center 1 week later due to rapid worsening of neurological symptoms with loss of mobility as well as bladder and bowel dysfunction.

Four months earlier, this patient had been hospitalized due to an mRNA-COVID-19-vaccine (Pfizer-BioNTech)-triggered corticosteroid- and immunoglobulin-refractory multiple autoimmune syndrome including (i) ADEM with severe left-sided atactic hemiparesis (MRC 2/5), contralateral dissociated sensory loss, and right-sided vestibulocochlear nerve deficit, and multiple acute inflammatory contrast-enhancing periventricular and right-sided brainstem lesions on magnetic resonance imaging (MRI), (ii) anti-acetylcholine receptor antibody positive ocular myasthenia gravis, and (iii) anti-thyroglobulin/anti-thyroid peroxidase/anti-thyroid stimulating hormone receptor autoantibodies positive thyroiditis. Due to symptom onset within 2–42 days after third dose of vaccine and exclusion of competing triggers, causal relationship of multiple autoimmune syndrome to vaccination could be assumed (9, 10). The patient had largely recovered after repeated PE and rehabilitation [see our previous case report (8)]. Importantly, multiphasic ADEM with two fully remitted episodes had been diagnosed 10 and 11 years ago, and family history was positive for Graves' disease by a daughter.

**Figure 1:**
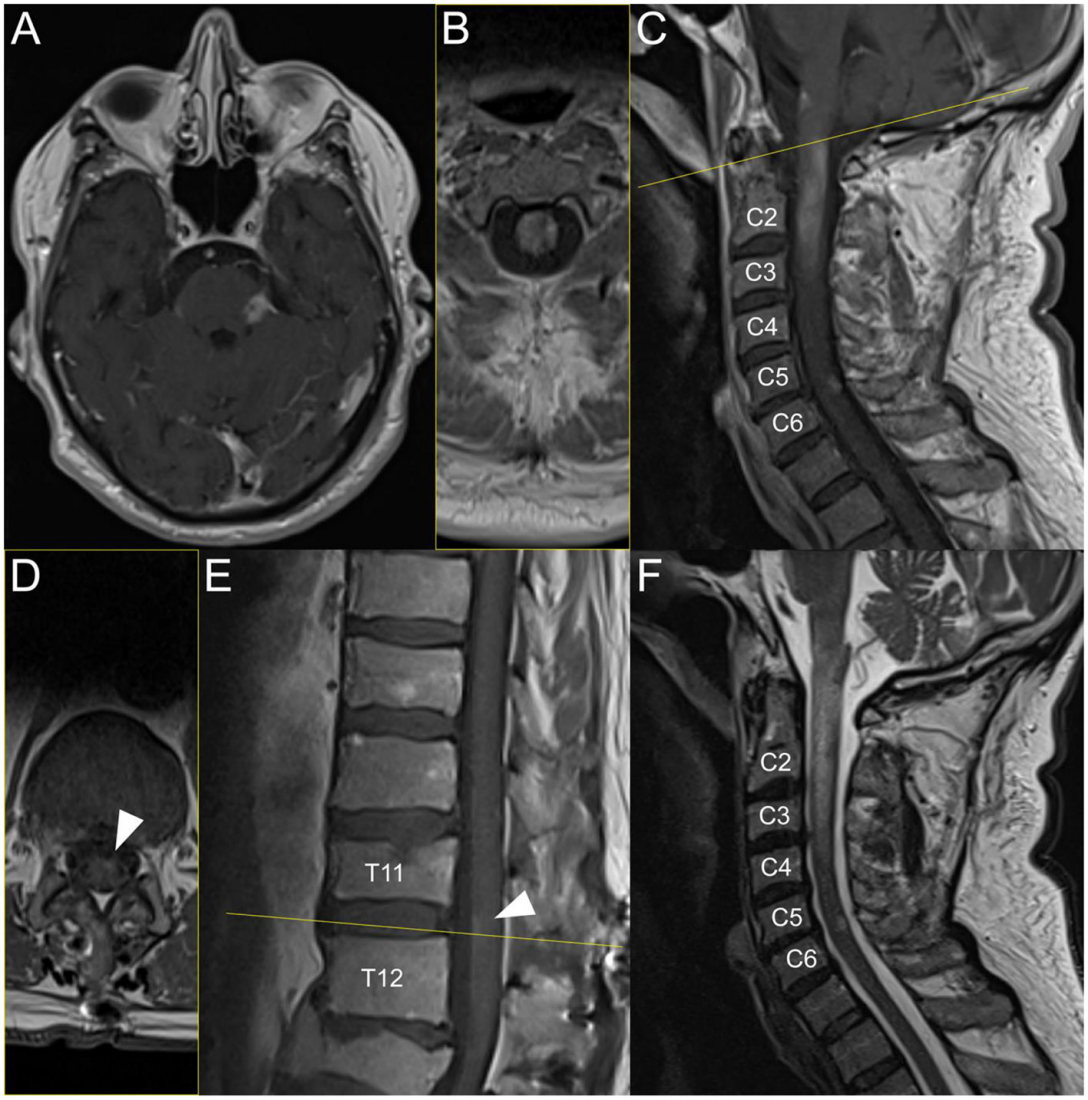
Brain and spinal MRI during post-SARS-CoV-2 infection-induced ADEM. Acute inflammatory contrast-enhancing T1 MRI lesions on the left middle cerebellar peduncle **(A)**, longitudinal extensive transverse myelitis from the lower part of the medulla oblongata to C3 **(C);** [panel **(B)** shows axial plane, i.e., yellow line on panel **(C)]** with edema lesion on T2 **(F)** reaching C4, on level C5/C6 **(C, F)**, and on the ventral conus **(E)**; [panel **(D)** shows axial plane, i.e., yellow line on panel **(E)**].

On current admission, the patient had severe spastic tetraparesis, sensory level C4, and urinary and fecal incontinence. MRI showed sequelae lesions of the previous vaccine-induced ADEM on the right cerebellar peduncle, right pons and right ventral medulla oblongata, but also new acute inflammatory contrast-enhancing T1 lesions on the left middle cerebellar peduncle, longitudinal extensive transverse myelitis from the lower part of the medulla oblongata to C3 (with edema T2 lesion reaching C4), on level C5/C6, and on the ventral conus (Figure 1). Cerebrospinal fluid (CSF) analysis showed mild lymphocytic pleocytosis (13 cells/μl), and a slightly increased protein level (64 mg/dl), while glucose and lactate levels were normal. The albumin ratio was elevated (10.7 × 10^−3^) and the immunoglobulin G (IgG) index was normal. Oligoclonal bands were the same in serum and CSF (type 4 pattern), and like during previous vaccine-induced ADEM, antibodies targeting antigens associated with demyelinating CNS disorders (i.e., myelin oligodendrocyte protein and aquaporin-4), and antibodies against extracellular and synaptic as well as intracellular neuronal antigens were negative. The inflammatory biomarker soluble interleukin-2-receptor was normal in CSF (< 50 U/ml), but slightly increased in serum (672 U/ml, normal range 158–613 U/ml). Screening for bacterial and viral neuroinfections was negative.

**Figure 2:**
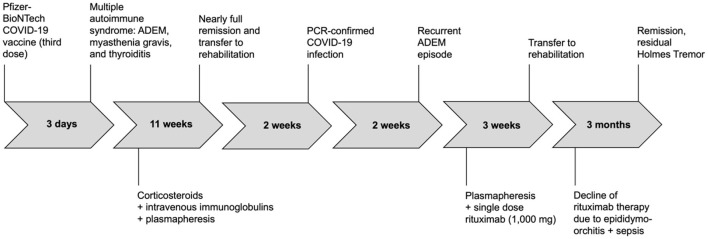
Timeline of clinical events and treatments.

Considering the significant response to PE during the vaccine-induced ADEM episode, we primarily opted for daily PE, which, unfortunately, did not improve symptoms this time. Therefore, we considered B-cell depleting therapy with rituximab. Following the first dose of 1,000 mg, the patient developed an epididymo-orchitis leading to severe sepsis and requiring orchiectomy. After recovery from infection and surgery, resumption of rituximab (or any other immunotherapy) was refused by the patient. The patient was then referred to rehabilitation. At 3-months follow-up, the patient had again largely recovered and was able to walk without any assistance. The only residual symptom was a Holmes tremor of his left arm, that was treated with levodopa. No follow-up brain MRI was conducted. Graus' criteria for definite ADEM were fulfilled for both previous vaccine-induced and current infection-triggered ADEM episodes (11). See Figure 2 for timeline of the case.

## SARS-CoV-2-RNA and -specific immunoglobulin G assessment

**Figure 3:**
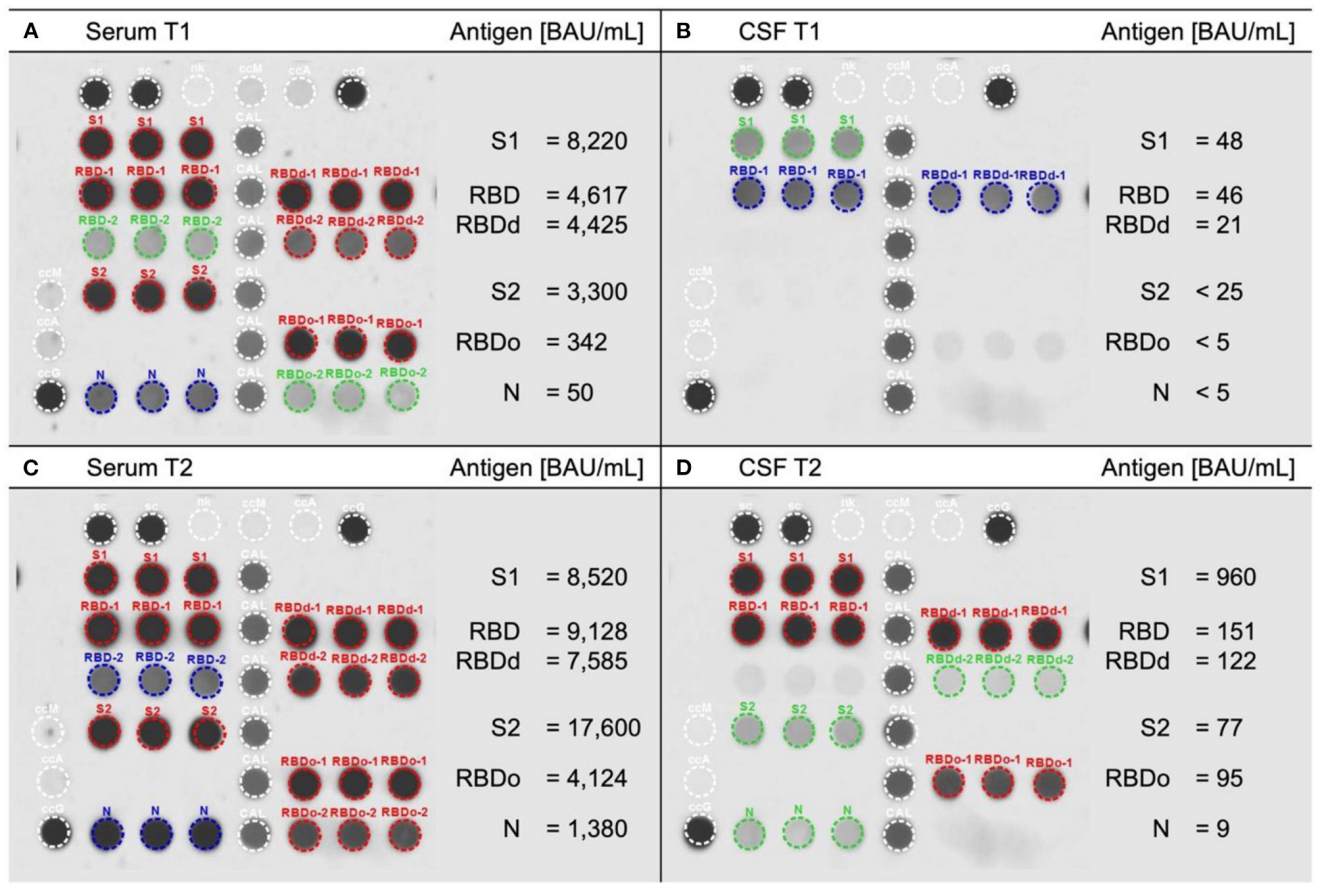
Measurement of antibodies against SARS-CoV-2 variants. Serum **(A, C)** and cerebrospinal fluid [CSF, **(B, D)**] collected during first vaccine-induced ADEM episode (T1, upper row) and during infection-induced ADEM episode (T2, lower row) analyzed using the SARS-CoV-2 variants of concern ViraChip^®^ immunoglobin G (IgG) test. This protein microarray detects IgG specific to SARS-CoV-2 surface [spike protein 1 (S1), receptor binding domains of the Wuhan (RBD), delta (RBDd), and omicron (RBDo) variants of concern, and spike protein 2 (S2)] as well as to the nucleocapsid antigen (N). RBDs are provided with high (RBD-1/RBDd-1/RBDo-1) and low (RBD-2/RBDd-2/RBDo-2) antigen concentrations. **(A)** Serum at T1 shows typical post-vaccine serum pattern with significant S1/RBD/RBDd/S2 but no N reactivity. **(C)** At T2, massive increase in serum reactivity including to RBDo and N confirms recent omicron variant infection. The synchronous mild increase in CSF reactivity from T1 **(B)** to T2 **(D)** proves blood-brain barrier patency of all surface and nucleocapsid IgG. BAU/ml, binding antibody units per milliliter; red/blue/green marking indicates high/moderate/low antigen-IgG reactivity.

Polymerase chain reaction collected from nasopharyngeal swab on admission at our center (28 days after diagnosis of SARS-CoV-2 infection) tested negative for SARS-CoV-2-RNA. Viral RNA in plasma and CSF was not tested. Detection of SARS-CoV-2 spike proteins and nucleocapsid in serum and CSF was performed using the SARS-CoV-2 variants of concern (VOC) ViraChip^®^ IgG test according to manufacturer's instructions (Viramed Biotech AG, Planegg, Germany). This protein microarray uses the purified surface proteins spike protein 1 (S1), receptor-binding domain RBD (Wuhan), RBDd (delta), and RBDo (omicron), as well as the nucleocapsid protein N of SARS-CoV-2 as antigens. Quantitative measurement of IgG-antigen binding was achieved using a World Health Organization international standard calibration. Serum and CSF samples, which were collected during the previous vaccine-induced ADEM episode (T1) and during the current infection-triggered ADEM episode (T2, 29 days after PCR-based diagnosis of SARS-CoV-2 infection) were analyzed (Figure 3). Serum reactivity constellation at T1 was well compatible with post-vaccination status (i.e., S1/RBD/RBDd/S2-IgG positive with no N-IgG). At T2, considerably increased levels of surface antigen-IgG including RBDo-IgG and additionally, high levels of N-IgG confirmed previous infection with the omicron variant of SARS-CoV-2. Whereas, S1/RBD/RBDd-IgG were detectable in CSF at both timepoints, with significantly higher concentrations after SARS-CoV-2 infection, RBDo-, S2-, and N-IgG were only detectable in CSF at T2 demonstrating recent omicron variant infection.

## Discussion

To our best knowledge, we report a first case with recurrent ADEM or ADEM-like disorder consecutively after SARS-CoV-2-mRNA-vaccination and SARS-CoV-2 infection. The case strongly demonstrates the possibility of both, post-vaccine and post-infection neuroinflammatory syndromes. Other inflammatory disorders of the CNS such as multiple sclerosis, paraneoplastic and other autoimmune or infectious encephalitis that represent the main differential diagnoses were excluded based on clinical, brain MRI, body CT, and CSF findings. Strongly suggesting genetic susceptibility of our patient, however, were two previous ADEM episodes more than a decade ago and the positive family history for autoimmune disease.

The mechanisms contributing to neurological manifestations in patients suffering from SARS-CoV-2 infection are still not fully elucidated, but have been associated to an excessive immune activation including the cytokine release syndrome (12). It is interesting to note that our patient showed an increased level of soluble interleukin-2-receptor in serum, which has been proposed as predictor of prolonged illness in SARS-CoV-2 infected patients (13). Nevertheless, it is not possible to establish a relationship with CNS involvement, since CSF levels were normal in our case. Moreover, we could detect anti-SARS-CoV-2-IgG in CSF, but while an increased albumin ratio as sign of blood-brain barrier damage was observed, specific CSF oligoclonal bands as well as an elevated IgG index indicating intrathecal production were absent. These observations agree with results reported in several previous studies, and emphasize that SARS-CoV-2 does not induce a specific intrathecal B-cell response but rather a systematic antibody production that may cross the blood-brain barrier (14). The lack of a large cytokine panel in serum and CSF as well as specific CNS biomarkers of neuronal injury (e.g., neurofilament light protein) in our case constitutes a limitation. The rarity with which SARS-CoV-2 was found in the CSF implies that the immune-mediated damage is more important than viral replication in neurons (15). Viral RNA in CSF was not tested in our patient. However, concentrations of anti-SARS-CoV-2-IgG in CSF were lower than those in serum, suggesting that the former originate from systemic source, either by passive diffusion across the blood-brain barrier or as a consequence of interactions between neurovascular units and viral components (15).

Furthermore, we had the opportunity to closely examine the serum and CSF antigenic profile of the patient during both events and observed, firstly, that particularly S2-specific IgG antibodies increased despite the absence of detectable viral RNA in the nasopharyngeal swab at the time of analysis. These data confirm previous reports that antibody assays may aid in diagnosing and staging of SARS-CoV-2 infections (16). Secondly, the negative S2-IgG in serum 3 months after third dose of SARS-CoV-2 vaccination (T1) provides further evidence for already suggested vaccine response kinetics and waning of humoral immunity (17). Last but not least, the observed massive increase of S2-IgG in serum asserts prior observations that natural infections after completion of vaccination induced a more robust immune response, characterized primarily by the stability of the IgG levels (18).

Considering the high concentrations of anti-SARS-CoV-2-IgG after third dose of vaccine and recent COVID-19 infection, a sufficient immunization could be assumed in our patient, and no further COVID-19 vaccination was discussed. However, we recommended monitoring of antibody levels, and pre-exposure prophylaxis with extended half-life monoclonal neutralizing antibodies against SARS-CoV-2 (19) in case of immune waning.

## Conclusion

Although the underlying mechanisms of post-SARS-CoV-2 infection-induced neuroinflammatory disorders are still not fully understood, the hypothesis that, similar to vaccine components, viral antigens may contribute to immune cell activation and CNS inflammatory response without direct viral implication, is strongly supported by our case.

Remarkably, all surface- and nucleocapsid-specific IgG in CSF followed a similar pattern compared to serum, however, with lower concentrations, which indicates their blood-brain barrier patency. Large CSF sample analyses are required to better understand pathogenesis in the CNS after SARS-CoV-2 infection and define treatment strategies.

## Data availability statement

The original contributions presented in the study are included in the article/supplementary material, further inquiries can be directed to the corresponding author.

## Ethics statement

Ethical review and approval was not required for the study on human participants in accordance with the local legislation and institutional requirements. The patients/participants provided their written informed consent to participate in this study.

## Author contributions

KP conducted the literature search and drafted the first version of the manuscript. SP supervised the study and made critical revisions. KH provided virological analyses. UE provided radiological expertise and MR images. MK, TI, and UZ made critical revisions. All authors contributed to the article and approved the submitted version.

## In memoriam

In memory of Professor Klaus Hamprecht. Virological analyses and support were provided by Professor Dr. Klaus Hamprecht, consultant virologist at the Institute of Medical Virology and Epidemiology of Viral Diseases at University Hospital Tübingen. We felt the loss of his thoughtfulness and thoroughness after his death.
